# Peripherally Induced Tregs – Role in Immune Homeostasis and Autoimmunity

**DOI:** 10.3389/fimmu.2013.00232

**Published:** 2013-08-07

**Authors:** Mahesh Yadav, Stephen Stephan, Jeffrey A. Bluestone

**Affiliations:** ^1^Diabetes Center, University of California San Francisco, San Francisco, CA, USA

**Keywords:** regulatory T cell, immune tolerance, autoimmunity, neuropilin-1, Helios

## Abstract

Thymically derived Foxp3^+^ regulatory T cells (tTregs) constitute a unique T cell lineage that is essential for maintaining immune tolerance to self and immune homeostasis. However, Foxp3 can also be turned on in conventional T cells as a consequence of antigen exposure in the periphery, under both non-inflammatory and inflammatory conditions. These so-called peripheral Tregs (pTregs) participate in the control of immunity at sites of inflammation, especially at the mucosal surfaces. Although numerous studies have assessed *in vitro* generated Tregs (termed induced or iTregs), these cells most often do not recapitulate the functional or phenotypic characteristics of *in vivo* generated pTregs. Thus, there are still many unanswered questions regarding the T cell receptor (TCR) repertoire and function of pTregs as well as conditions under which they are generated *in vivo*, and the degree to which these characteristics identify specialized features of pTregs versus features that are shared with tTregs. In this review, we summarize the current state of our understanding of pTregs and their relationship to the tTreg subset. We describe the recent discovery of unique cell surface markers and transcription factors (including Neuropilin-1 and Helios) that can be used to distinguish tTreg and pTreg subsets *in vivo*. Additionally, we discuss how the improved ability to distinguish these subsets provided new insights into the biology of tTregs versus pTregs and suggested differences in their function and TCR repertoire, consistent with a unique role of pTregs in certain inflammatory settings. Finally, these recent advances will be used to speculate on the role of individual Treg subsets in both tolerance and autoimmunity.

## Introduction

Immune tolerance is a key feature of the immune system that is designed to preserve self-tissues while allowing effective responses against infections. While most autoreactive T cells are deleted centrally in the thymus, peripheral T cells harbor self-reactive T cells that are kept in check by a number of intrinsic and extrinsic immunoregulatory mechanisms, among which suppressor or regulatory T cells (Tregs) play a crucial role. The importance of Tregs in maintaining peripheral tolerance to self-tissues is evidenced in both mice and humans by the fatal autoimmune disease that results from a loss of function mutation in the Foxp3 gene, the master transcription factor expressed selectively in Tregs (1–3). Tregs arise both in the thymus (tTregs) and extrathymically in the periphery (pTregs) as a consequence of induction of Foxp3 upon antigen exposure (4, 5). This nomenclature used to describe Treg subsets in this review is based on the recent recommendations by prominent researchers in the field (5). The discovery that TGF-β induces Foxp3 expression and suppressive activity in conventional T cells *in vitro* raised the possibility that Tregs could be extrathymically generated from naïve T cells in both mice (6, 7) and humans (8). However, signals that lead to the generation of pTregs *in vivo* have been less clearly defined. Historically, sub-immunogenic doses of antigen (9) as well as endogenous expression of foreign antigen in a lymphopenic environment (10) have been shown to induce pTregs *in vivo*. It is now becoming increasingly clear that pTregs arise in various conditions and could constitute a significant portion of Tregs in the periphery, especially in tissues such as the lamina propria (11). This also raises the question of whether pTregs are functionally similar to tTregs. Are pTregs induced to carry out a specific function or are they merely generated as a byproduct of antigen exposure in the periphery? Neonatal thymectomy experiments in mice strongly suggest that Tregs generated in the thymus are key to immune tolerance and peripherally generated Tregs are not sufficient to keep autoreactive cells in check (12–14). However, recently, pTregs have been shown to perform indispensable functions in controlling autoimmune responses under certain inflammatory conditions (15–17). With recent advances in the ability to distinguish thymic versus peripherally derived Tregs using neuropilin-1 (Nrp-1) and Helios, specific differences in gene expression, epigenetic modification, and the stability of Foxp3 expression between these two subsets are starting to emerge. Further defining their commonalities and differences will be important for elucidating biological functions and contributions of each Treg subset in maintaining peripheral tolerance, as well as their respective role in a variety of disease settings ranging from autoimmunity to cancer and infectious diseases. Of note, other subsets of Foxp3^−^ regulatory T cells with suppressor functions have been described, such as IL-10 producing Tr1 cells and TGF-β producing Th3 cells. However, in this review, we will focus on Foxp3^+^ tTregs and pTregs, highlighting key findings and recent progress in the field, and discussing the remaining unanswered questions.

## Characteristics of pTregs

Ever since it was discovered that TGF-β plays a key role in inducing Foxp3 expression in naïve T cells *in vitro*, there has been a considerable amount of interest in determining if a similar conversion of conventional T cells into Foxp3^+^ Tregs takes place *in vivo*. While Foxp3 is a critical orchestrator of Treg biology, it is not enough by itself to drive their full transcriptional program (18–21). Neither the induction of Foxp3 by TGF-β nor its exogenous expression by retroviral transduction can fully recapitulate the canonical Treg signature or the suppressive activities of tTregs (19, 20). Hence, *in vitro* generated iTregs may not replicate the true phenotype of *in vivo* peripherally generated Tregs and therefore are not ideal for studying pTregs.

Early evidence that pTregs were generated *in vivo* came from studies performed before the identification of Foxp3 as the master transcription factor for Tregs (22). Interestingly, pTregs in these studies were shown to exhibit a true Treg phenotype and to express canonical Treg markers such as CTLA-4, GITR, and CD103. Although the role of antigen exposure was not addressed in those studies, the requirement for IL-2 was clearly established. Later on, it was shown that optimal induction of pTregs is associated with non-immunogenic antigen delivery methods such as oral or intravenous injection, peptide pumps, or antibody-mediated DC targeting in the absence of adjuvants (9, 23). *In vivo* converted pTregs are effective suppressors in *in vitro* assays (9, 10, 24, 25) whereas TGF-β induced iTregs are not fully suppressive and acquire only a portion of the Treg transcriptional signature (6, 8, 19, 26) further highlighting the differences between iTregs and pTregs.

Feuerer et al. performed a comprehensive gene-expression analysis to characterize Foxp3^+^ Tregs generated under different conditions *in vivo*. Their analysis showed a remarkable heterogeneity between different populations, which perhaps highlighted the true adaptive nature of pTregs (20). Helios, an ikaros family transcription factor, was recently described as a specific marker for tTregs. Indeed, Thornton et al. reported that Helios is expressed highly on Foxp3^+^ Tregs in the thymus while approximately 70% of Tregs express Helios in the periphery (27). They suggested that these Helios^+^ cells may represent tTregs and that Helios could be used to distinguish between thymus- and periphery-generated Tregs. However, others have argued that Helios is induced during T cell activation and proliferation, and can also be upregulated in Foxp3^+^ iTregs *in vitro* and pTregs *in vivo* (28, 29). In addition to the these controversies, Helios is localized intracellularly and thus has a limited value as a marker to separate the two subsets of Tregs for functional studies.

We recently generated a myelin basic protein (MBP)-specific T cell receptor (TCR) transgenic (Tg) named 1B3 mouse in which pTregs were spontaneously generated in the periphery when these mice were crossed onto the RAG-2 knockout background. Through a series of experiments utilizing pTregs from this strain, we found that Nrp-1 was expressed on tTregs only and that Nrp-1 expression could be used to distinguish tTregs from peripherally generated pTregs in other settings (30). Consistent with the MBP.TCR.Tg 1B3 mouse, pTregs generated with low dose-antigen in ovalbumin-specific TCR.Tg BALB/c mice also failed to express Nrp-1, indicating that a lack of Nrp-1 expression is a general feature of pTregs. The Lafaille group reported similar findings where mucosa-generated pTregs expressed low levels of Nrp-1 in contrast to tTregs. This was further addressed in studies in mice lacking conserved non-coding elements at the Foxp3 locus (CNS1), the region that has binding sites for Smad 3 and the retinoic acid receptor. CNS1^−/−^ mice have normal numbers of tTregs but show severe impairment in the development of pTregs (16, 31). The frequency of Nrp-1^−^Foxp3^+^ Tregs is greatly reduced in the periphery in the CNS1^−/−^ mice, which is consistent with a lack of pTregs. The defects were most striking at mucosal surfaces, which are the primary sites for pTreg generation. Finally, Foxp3^+^ Tregs in the thymus express high levels of Nrp-1, although a small proportion of Nrp-1^lo^ cells are present among CD8^−^CD4^+^Foxp3^+^ cells. Not surprisingly, this subset is restricted to the CD24^hi^Qa-2^lo^ immature thymocyte subset, suggesting that tTregs upregulate Nrp-1 before they mature in the thymus (30). Weiss et al. further validated this finding in a series of experiments showing that Nrp-1^lo^ Foxp3^+^ cells in the thymus upregulate Nrp-1 before exiting the thymus (31). Of note, expression of Nrp-1 can distinguish pTregs and tTregs in circulating cells but not inflamed tissues since pTregs can upregulate Nrp-1 during inflammation, as discussed in the next section.

Epigenetic regulation of gene-expression plays an important role in differentiation and stabilization of T cell lineages (32, 33). In tTregs, demethylation of CpG islands in Foxp3 conserved non-coding region 2 (Treg-specific demethylation region or TSDR) is a hallmark feature and is thought to reflect stable, constitutive Foxp3 expression in this population (34). *In vitro* induction of Foxp3 by TGF-β is not sufficient to induce TSDR demethylation, whereas *in vivo* generated pTregs exhibit variable patterns. Some of the initial studies showed that *in vivo* generated pTregs have demethylated TSDR (35), although, this has been contradicted in recent studies showing that pTregs express methylated CpG motifs in TSDR (15). This, as discussed above, may reflect differences in the animal models used or perhaps may be due to the heterogeneity of the pTreg population. In our studies, pTregs that were isolated based on Nrp-1 expression show a pattern similar to tTregs with>85% demethylation in TSDR. Similarly, a recent study by Miyao et al. (36) showed that pTregs, once stabilized *in vivo*, display a demethylated TSDR (36). Recently, the Sakaguchi group further established that Treg development is contingent upon on CpG demethylation not only in the TSDR but also in signature genes such as Tnfrsf18, CTLA-4, Ikzf4, and Il2ra (37). Demethylation in these genes in tTregs establishes a tTreg-type CpG hypomethylation pattern, which is required for full Treg cell development in addition to Foxp3 expression. Interestingly, *in vivo* converted pTregs in their studies exhibited remarkable demethylation in the genes listed above, similar to what was observed in tTregs (37). Thus, Treg development is not solely dictated by the epigenetic regulation of Foxp3 but is achieved by the establishment of Treg-specific demethylation patterns and future epigenetic studies of pTregs need to include not only TSDR but also other signature genes to determine a fully committed Treg state.

## Are Tregs in Tissues Comprised Mostly of pTregs?

During inflammation Treg numbers increase in the relevant tissue and could constitute up to 50% of all CD4^+^ T cells. Tregs in tissues exhibit a unique phenotype that is reminiscent of the tissue microenvironment, as exemplified by PPARγ expression in Tregs in the adipose tissue (38). The term “tissular Tregs” has been used to define these tissue-resident Tregs (39). Tissular Tregs are not only important in controlling inflammation locally but also perform unique functions (which may be direct or indirect) such as controlling insulin sensitivity in the fat (40). Unpublished observations from our group further indicate that Tregs in tissues such as the muscle may be involved in tissue remodeling during an inflammatory or damage response (Villalta et al., unpublished observations). However, where these Tregs originate from is still unclear. They could arise in the thymus and accumulate in the tissue due to migration and proliferation in response to inflammation. Conversely, there is a strong possibility that these Treg cells are generated by conversion of CD4^+^CD25^−^ conventional T cells (Tconv cells) upon antigen encounter in the tissue. In support of this hypothesis, a recent study showed that tissue-resident macrophages play a key role in generation of pTregs in lungs. These macrophages coexpressed TGF-β and retinal dehydrogenases (RALDH1 and RALDH 2) under steady-state conditions and sampling of airborne antigens by these macrophages and presentation to antigen-specific CD4 T cells resulted in the generation of tissue-resident Foxp3^+^ Tregs (41). In ongoing studies in our lab, we have observed the accumulation of Tregs in muscles during inflammation. The origin of these Foxp3^+^ Tregs is still not known but they express high levels of Nrp-1 (Villalta et al., unpublished observations), suggesting their thymic origin. However, pTregs have been shown to upregulate Nrp-1 expression, especially in tissues during inflammation. Indeed, pTregs upregulate Nrp-1 during EAE or lung inflammation, and we also observed upregulation of Nrp-1 expression on pTregs during autoimmune response in pancreas (unpublished observations). Thus, Nrp-1-expressing Tregs present in inflamed tissues may not solely be thymically derived but could be generated by conversion. We believe that presence of pTregs could play a critical role in controlling local inflammatory responses in tissues and may have clinical significance for certain human diseases.

## Differences in pTreg versus tTreg Development

It has been postulated that tTreg development in the thymus is associated with high affinity TCR/MHC-peptide interactions while pTreg differentiation in the periphery is induced under sub-immunogenic conditions (9, 23, 31, 42, 43). This was evident in studies utilizing adoptive transfers of antigen-specific T cells, where the largest induction of Foxp3 in the periphery occurred after priming with low doses of their cognate antigen (44, 45). Interestingly, a low dose of high affinity agonist peptide supports pTreg induction while a low affinity peptide agonist poorly generates pTregs (46).

The relationship between the signaling pathways that promote the development of tTregs in the thymus and that elicit conversion into pTregs in the periphery is not entirely clear. TCR engagement and IL-2 signaling are indispensable for generation of all Tregs but pTregs require additional factors such as TGF-β and retinoic acid (47, 48). Blockade of TGF-β *in vivo* inhibits differentiation of antigen-specific pTregs (49). In mice lacking binding sites for smad3 in the Foxp3 enhancer region (CNS1), there is a lack of pTregs development (16). When congenically marked WT or CNS1^−/−^CD4^+^Foxp3^−^T cells were transferred into *RAG1*^−/−^recipient mice, the induction of Foxp3 was observed only in WT and not in the CNS1^−/−^ cells. Similarly, the *in vitro* assay demonstrated a significant reduction in the induction of Foxp3 in naïve T cells deficient in CNS1 (16) suggesting a dominant role for TGF-β signaling in extrathymic pTreg generation. It has also been argued that tTregs and pTregs have different requirement for co-stimulation. CTLA-4 has been shown to be upregulated on iTregs induced with TGF-β and its role in tTreg generation is debated (50, 51). In contrast, contribution of CD28 co-stimulation in tTreg generation in the thymus is well documented. The CD28-deficient mice show markedly lower number of Foxp3^+^ in thymus and the periphery (52, 53). CD28 may regulate Treg generation though alteration of avidity of T cell antigen-presenting cell (APC) interaction, promote IL-2 production or directly affect T cells signaling and survival (53, 54). However, whether CD28 is indispensable for pTreg generation has not been proven.

Besides these factors, pTreg generation in the periphery is thought to require self-antigen encounter by Tconv and may depend on encountering a specialized subset of APCs. As discussed earlier, APCs such as lung resident macrophages are conditioned by the local milieu and can develop the ability to induce pTreg conversion (41). In this regard, dendritic cells (DCs) are known to be highly tolerogenic in certain circumstances and their depletion can lead to decreased Foxp3^+^ Tregs and increased effector T cell responses, suggesting a major role for antigen presentation by DCs in maintaining/converting Tregs in the periphery (55– 57). Recent studies have led to the hypothesis that certain DC subsets are better equipped at converting Tregs than others. It was initially believed that antigen presentation by immature DCs leads to pTreg cell conversion whereas mature DCs promote effector function but more recent studies have questioned this (57– 59). Targeting of antigen to immature DCs via DEC205 or antigen presentation by CD103-expressing DCs favor the induction of pTregs *in vivo* (10, 48, 60). A recent report showed that migrating DCs are superior to tissue-resident DCs in their ability to induce Foxp3 (61). In this study by targeting self-antigen to skin migratory or lymphoid-resident DCs the investigators found that skin langerin^+^ DCs have unique ability to promote generation of pTregs *in vivo*. Moreover, there is evidence that plasmacytoid DC subsets can also enhance induction of pTregs in mucosal sites such as the lung (62). Hence, the combination of soluble factors in the microenvironment, such as TGF-β and IL-2, and antigen presentation by specialized APCs seems to be critical for pTreg cell generation. This is particularly evident in the gut mucosa, where pTregs are generated with precise antigen specificities and characteristics. This results in a specialized pTreg subset, which is important for controlling local inflammatory responses but differ functionally from the tTregs that are generated to maintain general immune homeostasis. In this regard, the specific contribution of individual APC subsets to Treg induction in the thymus is not completely understood. Although it has been shown that antigen presentation by AIRE expressing medullary epithelial cells and DCs are important in Treg differentiation, the pathways involved are still poorly defined (63–65). Recently, the CD27-CD70 pathway has been shown to be important in promoting Treg development by DCs and medullary epithelial cells (66). CD70 expression on medullary thymic epithelial cells and on DCs enhanced positive selection of Tregs and promoted the survival of developing Tregs. Of note, AIRE expressing extrathymic cells have been described as regulating peripheral tolerance but whether this is mediated through pTreg induction has not been addressed (67).

## Function and Stability of pTregs

**Figure 1:**
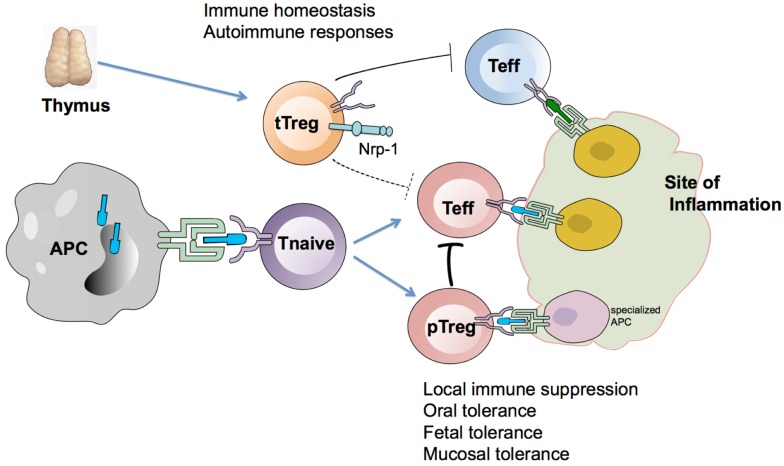
**Model depicting the generation and function of tTregs and pTregs**. Nrp-1^hi^ tTregs are generated in the thymus and are important in maintaining immune homeostasis and controlling autoimmune responses. During the course of an immune response, Nrp-1^lo^ pTregs are generated in response to Ag presentation by specialized APCs and control effector T cells (Teff) at the site of inflammation. pTregs help in controlling inflammation locally and may be more effective than tTregs at suppressing Teff due to overlapping antigen specificity.

It is well established that tTregs are crucial for preventing autoimmunity and exaggerated immune responses. Thymectomy in mice on day 3 after birth results in organ-specific autoimmune diseases due to lack of Treg development, which can be prevented by inoculation of CD25^+^CD4^+^ Tregs (13, 14). These findings suggest a limited role for pTregs in the absence of tTregs in controlling autoimmune responses. However, studies aimed directly at analyzing pTregs function *in vivo* have been few, due to the lack of appropriate animal models. Most functional studies have utilized *in vitro* TGF-β-induced iTregs and have shown them to be protective (25, 68, 69). In this regard, TGF-β-induced antigen-specific iTregs are highly efficient in controlling onset of autoimmunity in murine model of autoimmune gastritis through inhibition of DC functions and modulation of T cell trafficking (70, 71). However, studies comparing suppressive functions of Treg subsets directly, have found iTregs to be less efficient than tTregs (15, 19). These studies likely reflect a lack of acquisition of the full Treg program by TGF-β-induced iTregs, which in combination with other factors, such as number of cells injected and type of animal model used, may influence their efficacy. The functional analysis of pTregs has mostly been limited to mucosal tolerance, inflammatory responses to foreign antigens, and animal models that may not reflect physiological conditions. Haribhai et al. showed recently that tTregs were unable to suppress chronic inflammation and autoimmunity in the absence of pTregs (15). In their model, tTregs alone were not sufficient to maintain tolerance when transferred into Foxp3-deficient mice. However, when Foxp3^−^ Tconv cells were co-injected with tTregs, peripherally generated pTregs represented ∼15% of Treg pool and acted in concert with tTregs to restore tolerance. It is difficult to draw full conclusion based on these studies due to the reported inconsistencies in the behavior of effector T cell responses in scurfy mice. Despite this, if similar functions of pTregs were observed in other animal models, it would support an interesting paradigm, that pTregs are generated to complement tTregs and contributions by both pTregs and tTregs are necessary to establish tolerance. We further hypothesize that tTregs are required for immune homeostasis and broad-spectrum control of autoimmune responses, whereas pTregs are generated to control inflammation locally in tissues and this suppression may be transient due to the short lifespan/stability of pTregs (Figure 1). In this regard, the Rudensky group has argued that pTregs have a limited role in maintaining tolerance by showing that the absence of pTregs does not result in spontaneous autoimmunity or exacerbation of induced tissue-specific autoimmunity. They used CNS1^−/−^ mice, which have selective impairment in pTreg generation, and showed that CNS1^−/−^ mice developed pronounced Th2-type pathologies with hallmarks of allergic inflammation and asthma (16). This was attributed to a lack of GATA-3-expressing Tregs in CNS1^−/−^ mice, in agreement with recent studies showing that Tregs can specifically suppress immune responses driven by a given effector T cell subset (Th1, Th2, etc…) by expressing transcription factors and chemokine receptors typically associated with this subset. Although consistent expression of Foxp3 is required to reinforce the regulatory program, Treg cells can also undergo stimulus-specific differentiation that is regulated by transcription factors typically associated with the differentiation of conventional CD4^+^ T cells. This results in effector Tregs with unique migratory and functional properties expressing transcription factors involved in regulation of the corresponding type of effector immune responses. These “effector Tregs” have unique functional properties and are better equipped to control ongoing immune responses (72, 73). The first evidence of effector Tregs came from findings showing that the expression of IFN regulatory factor (IRF) 4, which is required for the differentiation of Th2 and Th17 cells, is required for the control of Th2-driven autoimmunity (74). This concept has further been extended after subsequent studies showing T-bet and STAT3 expression in Tregs control Treg migration and suppressive functions during Th1 and Th17 immune responses, respectively (75, 76). Hence, in CNS1^−/−^ mice, the lack of GATA-3^+^ Tregs could be responsible for the exaggerated Th2 response. This raises an interesting possibility that effector Tregs are part of the pTreg pool, which allows them to be better equipped with effector T cell machinery. This possibility has not been addressed directly. One of the most prominent functions of pTregs has been reported in the maintenance of fetal tolerance during pregnancy. During pregnancy, pTregs are generated against a paternal alloantigen in a CNS1 dependent manner and enforce maternal-fetal tolerance. CNS1 deficient females exhibit increased embryo resorption accompanied by increased immune cell infiltration during allogeneic but not syngeneic pregnancy, which are features observed in human preeclampsia (17). A similar phenomenon has been observed in human pregnancy, where Helios^−^Foxp3^+^ Tregs are increased in the peripheral blood of healthy pregnant women when compared to non-pregnant controls or preeclamptic patients (77). These results argue that pTregs serve as the predominant subset in suppressing the fetal-specific immune response and defect in pTregs may be central to pathogenesis of preeclampsia (78, 79).

In our studies, we found that pTregs were efficient in controlling the islet-specific autoimmune response in lympho-replete conditions in NOD.CD28^−/−^ mice, which have a greatly reduced number of tTregs (30). In contrast, in lymphopenic conditions, we found that Nrp-1^hi^ tTregs were able to control EAE induced by MBP-reactive T cells but Nrp-1^lo^ pTregs were unable to exhibit similar suppressive functions *in vivo*. These results suggest that the functions of pTregs and tTregs are not overlapping and these subsets may present specialized suppressive functions adapted to individual immunological milieus and inflammatory settings. Nrp-1 is a key protein with important functions in Tregs that may provide Nrp-1-expressing tTregs a functional superiority over pTregs. Indeed, Nrp-1 can enhance the interactions between Treg cells and DCs and can directly promote the activation of the latent form of TGF-β (80, 81). It remains to be explored whether reduced expression of Nrp-1 on pTreg cells result in compromised suppressive function under certain inflammatory conditions. In this regard, it has been shown that Treg cells from Nrp-1^−/−^ mice are less suppressive than WT Treg cells and blocking of Nrp-1 abrogates suppression of proliferation of responder T cells by Treg cells (81).

**Figure 2:**
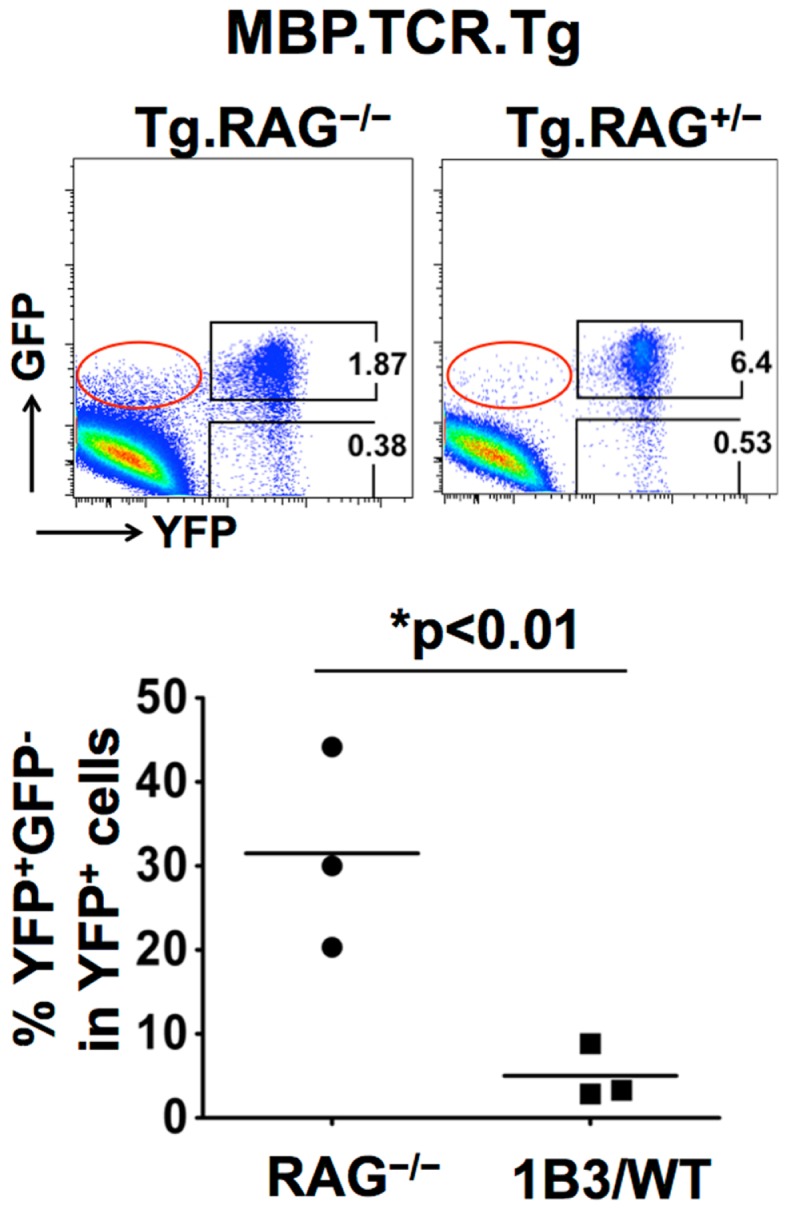
**Stability of Treg subsets in MBP.TCR.Tg 1B3 mice using lineage reporter system**. The MBP.TCR.Tg mouse when crossed onto RAG^−/−^ background lacks tTregs but generates pTregs in the periphery highlighted by red box (GFP^+^YFP^−^ subset). The FACS plots depicting expression of GFP and YFP by CD4^+^ T cells from LNs of 3- to 4-week-old MBP.TCR.Tg.RAG^−/−^.Foxp3-Cre × R26-YFP or MBP.TCR.Tg.RAG^+/−^. Foxp3-Cre × R26-YFP mice are shown. In the current gating strategy, GFP^+^YFP^+^ population represents the stable Treg subset whereas GFP^−^YFP^+^ gate represents unstable Tregs, which previously expressed Foxp3. Cells gated on CD4^+^ T cells are shown and numbers around the outlined areas indicate percent. Graph on bottom shows the frequency of GFP^−^YFP^+^ among YFP^+^ cells with each symbol representing an individual mouse and bars representing mean values for each group.

One of the striking differences we observed between two subsets of Tregs was the stability of Foxp3 expression. Under lymphopenic conditions, where IL-2 availability might be limited, a greater proportion of pTregs lost Foxp3 compared to tTregs. This was also evident when the MBP.TCR.Tg 1B3 mouse was crossed onto a Treg lineage reporter system. 1B3.RAG^−/−^ mice, which develop Tregs only in the periphery, lack Tregs in the thymus. In order to lineage track Tregs we crossed MBP.TCR.Tg 1B3 mouse onto Foxp3.GFP.Cre.YFP^fl/fl^ background (82), there was a significant increase in the frequency of YFP^+^GFP^−^ “exFoxp3 cells” compared to WT or 1B3.RAG^+/−^ mice (Figure 2). Decreased stability and plasticity of Foxp3 expression in pTregs is perfectly in line with the overall function of pTregs, i.e., to control ongoing inflammation and then decline once immune responses are terminated (83–85). The instability of Foxp3 expression in pTregs may allow these cells to revert back to Tconv cells once the inflammation is cleared or antigen presentation is reduced, helping them in responding to a local inflammation without having a long-term suppressive outcome. This notion was further supported in studies by Miyao et al. showing that peripherally induced Foxp3^+^ T cells contain both unstable and stable cells which show reduced stability compared to tTregs in lymphopenic conditions (36). Thus, the growing evidence suggests that while tTregs are central to immune homeostasis and controlling autoimmunity, pTregs have specialized functions depending on the type of inflammation while playing an indispensible role in certain settings such as mucosal immunity and fetal tolerance.

## Differences in the TCR Repertoire of pTregs and tTregs

It is now well accepted that TCR diversity plays a crucial role in thymic selection and also differentiation of Tregs. During T cell development in the thymus, an extremely diverse set of TCRs is selected into the peripheral repertoire during a process in which thymocytes with highly reactive TCRs that potentially see self-antigens are eliminated while cells with intermediate affinity TCRs are selected into Tregs. The Treg repertoire is highly diverse with a wide range of antigen specificities but marked reactivity to self-antigens, and very little overlap with the repertoire of Tconv cells (86–88). Although the affinity of TCRs expressed by Tregs for self-antigenic peptide/MHC complexes remains to be fully defined, it is believed to be 100-fold lower than negatively selected TCRs. Only a handful of studies have tried to address the shaping of the TCR repertoire in pTregs compared to tTregs, partly because of the paucity of an appropriate model to generate pTregs *in vivo*. The “division of labor” concept for the pTreg and tTreg populations would suggest a limited clonal relationship between these two subsets. In this regard, studies of TCR repertoire in Tregs from the intestinal mucosa, which comprises mostly pTregs (discussed in details later), have provided some useful insights (89). Indeed, Tregs isolated from the gut express TCRs that appear different from those used by Tregs in other locations, implying that pTregs in the gut have a distinct repertoire that may be shaped by interactions with local antigens.

**Figure 3:**
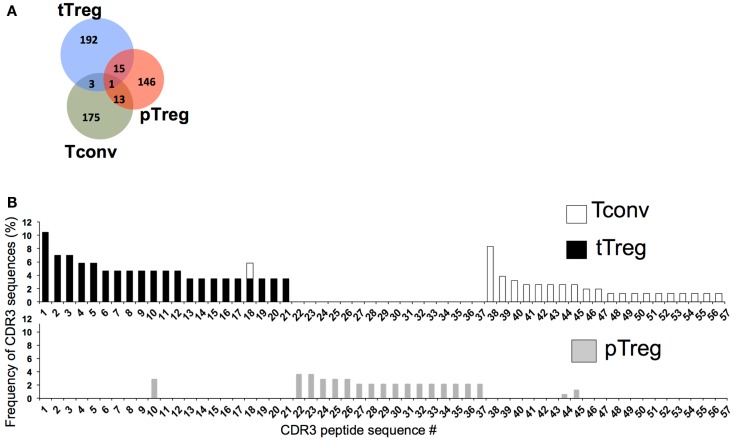
**Distinct TCR repertoire of pTregs and tTregs**. **(A)** Venn diagram showing distribution of unique and overlapping pTreg, tTreg and Tconv CDR3 sequences. Nrp-1^hi^ tTregs, Nrp-1^lo^ pTregs and CD4^+^Foxp3^−^ Tconv cells were sorted from MBP.TCR.Tg (1B3)-Foxp3.GFP mice. cDNA was amplified with Vα2-specific primers and amplicons were subcloned and sequenced to determine CDR3 sequences. **(B)** Frequency of unique CDR3 sequences (identified by peptide number along horizontal axis) in Nrp-1^hi^ tTregs (black bars; top graph), Foxp3^−^ Tconv cells (white bars; top graph) and Nrp-1^lo^ pTregs (gray bars; bottom graph) sorted from 1B3 mice. Data from one representative mouse is presented here.

We took advantage of our ability to separate Tregs into different subsets based on Nrp-1 expression to compare the TCR repertoire of Nrp-1^hi^ and Nrp-1^lo^ Treg subsets using complementarity-determining region 3 (CDR3) sequencing. To limit the overall diversity of the repertoire for this study, we used MBP.TCR.Tg.Foxp3-GFP mice and isolated Nrp-1^hi^ tTregs, Nrp-1^lo^ pTregs, and Tconv (CD4^+^Foxp3^−^) cells, then amplified, cloned, and sequenced a region of the α chain encompassing the CDR3 for Vα2^+^ TCR. Although it was a limited analysis and may not represent the whole repertoire, it still provided useful information. Out of nearly 290 clones per subset, 175 pTregs, 212 tTregs, and 192 Tconv cells had productive V–J rearrangements. CDR3 amino acid sequence analysis of Vα2 revealed that there was limited overlap between the tTreg and pTreg CDR3 sequences (Figure 3). The pTreg subset shared only 8 and 9.1% CDR3 amino acid sequences with Tconv cells and tTregs, respectively. As shown in Figure 3, the most frequent CDR3 sequences detected in tTreg and Tconv cells were rarely used in pTregs. The limited overlap between the pTreg and tTreg subsets was expected and is consistent with other recent studies using peripherally generated pTregs (15, 89) and again emphasized the different lineage development of Nrp-1^hi^ tTregs and Nrp-1^lo^ pTregs. Interestingly, very few of the TCRs sequences overlapped with Tconv TCRs as well suggesting that the pTregs represent a very small, presumably self-antigen-specific TCR subset within the large Tconv repertoire. This is consistent with recent findings by others (89) and fit with the notion that distinct TCR ligand affinity may dictate the generation of pTregs in the periphery. However, our analysis was limited to a relatively small number of TCR sequences and a more thorough repertoire analysis of Nrp-1^hi^ and Nrp-1^lo^ Tregs is needed to further substantiate these findings.

Having distinct sets of TCRs in pTregs allows Tregs to have a broader repertoire overall, which is important for recognition of a wide array of potential self and foreign antigens and ensures that Tregs can play their role in a large variety of immune responses. Although it remains unclear if cells expressing certain TCRs are more disposed to turn on Foxp3 in the periphery. It is well known that TCR affinity required for tTreg development is higher than that required for positive selection of Tconv cells and lower than for negative selection. Interestingly, the lack of overlap in the repertoire of pTregs and tTregs (Figure 3) suggests that the TCRs of Tconv cells that turn on Foxp3 in the periphery evade being selected on tTregs in the thymus. This indicates that antigen encounter by a TCR has different outcome in the periphery versus the thymus. One possibility that has been raised recently is that antigenic peptides may bind in more than one register to the MHCII and this may affect interactions with the TCR. Poor binding, or binding in a different register, may prevent thymic deletion and allow autoreactive T cells targeting self-antigens to escape negative selection. For instance, a segment from the insulin beta chain (B:9–23), which is a major target of autoreactive CD4^+^ T cells in humans and NOD mice (90), can bind the groove of NOD MHC I-Ag7 molecules in at least three overlapping adjacent registers. Most B:9–23 specific CD4^+^ T cells in the periphery recognize peptides bound in an unusual and not predicted register due to the poor affinity for MHC class II binding (91–95). These unique registers of insulin peptides may be generated by processing of insulin and peptide loading into I-Ag7 molecules that occur specifically in the pancreas and are distinct from the classical APCs (94, 96). Thus, the fact that functional peripheral registers display weak binding to I-Ag7 molecules and/or are generated exclusively in the periphery may explain how T cells specific for these peptides can escape thymic negative selection in NOD mice. In the periphery, uptake and processing of tissue-derived proteins and peptides by a different type of APCs could give rise to peptide-MHC complexes in a distinct register, which are not found in the thymus and could result in high affinity interactions with the TCR that trigger the activation of T cells. Such interactions have been show to turn on Foxp3 in naïve T cells in the periphery and participate in induction of peripheral tolerance (97). Thus, presentation of antigen differently in the periphery for Foxp3 induction could be one of the ways by which a broader TCR repertoire in the Treg pool is achieved.

## Role of the Gut Microbiota in Inducing pTregs

Several studies have shown that the frequency of Foxp3^+^ Tregs among CD4^+^ T cells is notably higher, due to peripheral conversion, in mucosal surfaces than in other tissues (48, 49, 60). In humans,>100 trillion bacteria, which represent over 100 different species, colonize the skin and mucosal surfaces, including the oral cavity and the intestine. This leads to a complex ecosystem with continuous interplay between host cells and the microbiota. Many studies have shown that the intestinal mucosa is a preferential site for the peripheral induction of Tregs, suggesting that the high frequency of mucosal Tregs may be due to this locally superior conversion into pTregs. Consistent with this hypothesis, the frequency of Nrp-1^lo^Foxp3^+^ Tregs is increased in the colonic lamina propria. Another non-exclusive possibility is that the increased frequency of pTregs in the presence of commensal bacteria in the gut microenvironment is partly due to selective survival of Nrp-1^lo^ pTregs. Interestingly, colonic Treg numbers are greatly reduced in germ-free mice, suggesting the dependence of gut Tregs on the commensal microbiota (98–100). However, the exact mechanisms by which Tregs are generated in response to self-antigens or foreign antigens derived from commensal bacteria remain unclear. Bacterial metallo-matrix proteases could potentially contribute to the conversion of TGF-β to its active form and thus participate in induction of pTregs in the gut (101). There is a growing amount of literature suggesting that the development of T cell subsets, including Tregs, is influenced by a single species of microbe in the gut (101–104). Indeed, colonization by the bacterium *Clostridium*, or *Bacillus fragilis*, leads to induction of Foxp3 expression in Tconv cells (101). The *Clostridium* species is indigenous and provides a TGF-β-rich environment that may facilitate the induction of Foxp3 in colon. While *B. fragilis* is a human commensal, it could increase the frequency of colonic Tregs when it colonized the mouse gut by means of a protease-resistant capsular polysaccharide. Polysaccharide A from *B. fragilis* can also act directly on Tregs through TLR2 (104). Although this field is still in its early stages, these findings may result in development of novel ways of inducing tolerance through colonization of a single species in the gut, which could be useful in inflammatory bowel disease or other indications requiring the generation of pTregs. The induction of Tregs in the gut is also influenced by the presence of APCs specialized in picking up bacterial products and presenting them to T cells. CD103-expressing DCs are present in abundance in the gut and are specialized in inducing Treg differentiation from naïve CD4^+^ T cells (48, 60). These migratory DCs are responsible for picking up bacterial pathogens from the intestinal epithelium and transporting them to the lymph nodes to present antigens to T cells (105, 106). In addition, CD11b^+^ lamina propria macrophages express retinoic acid dehydrogenase and are able to induce the differentiation of Tregs in the intestine (107). Thus, gut microbes can initiate the generation of pTregs in the gut in many different ways.

## Importance of Separating Subsets of Tregs in Humans

We have learned a great deal about the functions of Foxp3^+^ Tregs through studies of murine Tregs. However, in light of current and future clinical applications of Tregs, it is imperative to define the subsets of human Tregs and how they relate to mouse Tregs. The extent to which pTregs are represented in the peripheral pool in humans is controversial, and differences observed between Tregs in humans and mice could hamper our ability to translate findings on murine pTregs to pTregs in humans. For example, a vast majority of circulating human Tregs express CD31, a marker for recent thymic emigrants, thus suggesting their thymic origin (108). However, when we analyzed in humans the expression of Nrp-1, a marker for murine circulating tTregs as discussed above, we could not detect Nrp-1 expression on human Tregs in the peripheral blood whereas greater than 70% of circulating Tregs express Nrp-1 in mice. This is consistent with earlier reports with exception of one study in which Nrp-1^+^ Tregs were detected in human lymphoid organs (109, 110). We also analyzed healthy human splenic Tregs and found little or no expression of Nrp-1 on Foxp3^+^ cells (unpublished observations). Another significant difference between human and mouse T cells is that human Tconv cells can express Foxp3 upon transient activation more readily than mouse cells (111–113). Whether Foxp3 expression in activated cells is (111, 114) or is not (115–117) associated with acquisition of suppressor function remains controversial. However, because human T cells can express Foxp3 upon activation even in the absence of TGF-β (118), it makes it harder to distinguish activated T cells from pTregs even though Foxp3^+^ activated T cells do not exhibit any of the canonical markers of human Tregs.

Among other markers found on mouse Tregs, Helios expression has correlated very well in humans Tregs. Like mouse, Helios is expressed highly on human Tregs with greater than 70% of Foxp3^+^ Tregs expressing Helios in the peripheral blood. Although the Helios^−^Foxp3^+^ Tregs fit the profile of pTregs, whether these cells originate outside thymus remains controversial. Among other evidences, effector cytokine secretion, which has been proposed to be a function of pTregs, has been associated with Helios^−^Foxp3^+^ Tregs (119, 120). Conversely, a recent study made the argument that both Helios^−^ and Helios^+^ Tregs are of thymic origin by showing that both Helios^−^ and Helios^+^ Tregs exhibit a demethylated TSDR and express canonical Treg markers such as CD39 and CTLA-4 in human peripheral blood (121). However, Helios^−^ Tregs were gated on the CD45RA^+^ naïve Treg population in that study and may not include the whole Helios^−^Foxp3^+^ T cell population. In addition, studies in mice have shown that pTregs are very similar to tTregs in terms of TSDR demethylation and expression of Treg canonical markers, raising the possibility that these parameters may not adequately discriminate the pTreg and tTreg subsets in humans as well. It has also been argued elsewhere that Helios can be expressed in conventional human T cells upon activation (28). In this regard, Nrp-1 expression is upregulated in iTregs in mice and pTregs can also upregulate Nrp-1 expression during inflammation in tissues suggesting similar upregulation of Helios could be happening on human T cells. Hence, despite the controversies Helios remains the best marker to separate tTregs from the peripherally generated pTregs in the human peripheral blood. In our laboratory, we recently identified a subset of Foxp3^+^ Tregs in humans that also expressed IFN-γ. Despite high levels of Foxp3, these IFN-γ^+^Foxp3^+^ cells lack Helios expression and show a partially methylated TSDR in the Foxp3 locus, and therefore fit the profile of peripherally generated Tregs. Moreover, since Helios is selectively expressed on IFN-γ^−^ Tregs (119), ongoing studies in our laboratory aimed at characterizing these cells may facilitate the identification of a putative surface marker for Helios-expressing Tregs that will more reliably separate tTregs from other pTreg subsets.

Lastly, understanding Treg subsets in humans is also important because Treg dysfunctions have been reported in several human autoimmune diseases (119, 122–125). We learned from mouse studies that pTregs are less stable than tTregs and may have compromised functions in certain inflammatory conditions, notably in the autoimmune setting. Whether a similar defect in pTreg population leads to Treg dysfunction in autoimmune patients remains to be seen. It would be possible to address these issues once the markers for human pTregs are defined. This may also have important repercussions on immunotherapies designed to restore Treg-mediated tolerance in diseases where targeting tissues are not readily accessible for functional studies, such as type 1 diabetes or multiple sclerosis. Using mouse models that mimic the human immune system may also help approach some of these questions. In this regard, humanized mice generated using cord blood or transplantation of human thymus and bone marrow cells could prove useful and may help resolve some of these issues.

## Concluding Remarks

Ever since the discovery of CD25 and Foxp3 as markers of regulatory T cells, there has been some controversy in the field regarding the existence and development of pTregs. New technologies in gene profiling, cell sorting, and mouse engineering have made it clear that pTregs develop under normal homeostasis as well as under inflammatory conditions. Identification of genes that are differentially expressed between Treg subsets and mouse models of pTreg generation have helped in differentiating characteristics of pTregs from tTregs. Functionally, the role of pTregs in mucosal tolerance is already pretty well established, and it is now becoming increasingly evident that these Tregs have specialized functions in response to non self-antigens during conditions such as asthma and fetal tolerance. However, a number of key questions still remain. What are molecular determinants that contribute to the induction of Foxp3 in the periphery? Is Foxp3 induction in the periphery restricted to a subset of Treg precursors? How does TCR affinity or strength of signal influence Treg generation in the thymus versus periphery? What are the different conditions under which pTregs play an indispensable role? Finally, how can we utilize pTregs to improve Treg therapy in human conditions? The definition of new markers to complement Nrp-1 and Helios and new mouse models and humanized mouse models of pTreg generation will undoubtedly play a part in answering some of these questions in the very near future. A better understanding of the biology of pTregs will in turn provide a clearer view of the respective role of pTregs versus tTregs in a number of human pathologies and will be important in devising optimal therapeutic strategies as Tregs are increasingly being considered as either tools or targets of immunotherapy in many diseases.

## Conflict of Interest Statement

The authors declare that the research was conducted in the absence of any commercial or financial relationships that could be construed as a potential conflict of interest.
